# Plasma Brain-Derived Neurotrophic Factor Levels Predict the Clinical Outcome of Depression Treatment in a Naturalistic Study

**DOI:** 10.1371/journal.pone.0039212

**Published:** 2012-06-27

**Authors:** Masatake Kurita, Satoshi Nishino, Maiko Kato, Yukio Numata, Tadahiro Sato

**Affiliations:** 1 Sato Hospital, Koutokukai, Nanyo, Yamagata, Japan; 2 Department of Cellular Signaling, Graduate School of Pharmaceutical Sciences, Tohoku University, Sendai, Miyagi, Japan; University of Nebraska Medical Center, United States of America

## Abstract

Remission is the primary goal of treatment for major depressive disorder (MDD). However, some patients do not respond to treatment. The main purpose of this study was to determine whether brain-derived neurotrophic factor (BDNF) levels are correlated with treatment outcomes. In a naturalistic study, we assessed whether plasma BDNF levels were correlated with clinical outcomes by measuring plasma BDNF in patients with depressive syndrome (MADRS score ≥18), and subsequently comparing levels between the subgroup of patients who underwent remission (MADRS score ≤8) and the subgroup who were refractory to treatment (non-responders). Patients with depressive syndrome who underwent remission had significantly higher plasma BDNF levels (*p*<0.001), regardless of age or sex. We also found a significant negative correlation between MADRS scores and plasma BDNF levels within this group (ρ = –0.287, *p* = 0.003). In contrast, non-responders had significantly lower plasma BDNF levels (*p* = 0.029). Interestingly, plasma BDNF levels in the non-responder group were significantly higher than those in the remission group in the initial stage of depressive syndrome (*p* = 0.002). Our results show that plasma BDNF levels are associated with clinical outcomes during the treatment of depression. We suggest that plasma BDNF could potentially serve as a prognostic biomarker for depression, predicting clinical outcome.

**Trial Registration:**

UMIN Clinical Trials Registry UMIN000006264

## Introduction

Remission is defined in The Diagnostic and Statistical Manual (DSM-IV) as the absence of significant signs or symptoms, and is the primary goal of treatment for major depressive disorder (MDD). At present, the category of depression (e.g., severe, moderate, mild or remission) is primarily evaluated by the patient using various subjective indices; however, there is currently no biomarker that could serve as an objective index for evaluating the severity or progression of MDD.

Brain-derived neurotrophic factor (BDNF) has increasingly attracted attention among researchers investigating MDD, as numerous reports have indicated that it plays an important role in the illness [1]. BDNF is a member of the neurotrophin family and plays a critical role in the survival, differentiation and outgrowth of peripheral and central neurons during development and in adulthood [2], [3].

Serum and plasma levels of BDNF are decreased in patients suffering from MDD [4], [5], [6], [7], [8]. In addition, serum BDNF levels are correlated with the severity of depression [9], and serum BDNF levels in patients treated with antidepressants increase to levels found in healthy subjects [8], [10], [11]. Moreover, BDNF levels in the hippocampus and prefrontal cortex are significantly reduced in suicide victims compared with non-suicide controls [12]. Although most studies to date have shown that serum BDNF levels increase with antidepressant treatment, the usefulness of serum BDNF as a biomarker for MDD is not yet clear.

Among MDD patients, there exist two distinct groups: a group that responds to treatment (the responder group) and a group that is refractory to treatment (the non-responder group). To our knowledge, no study to date has examined plasma BDNF levels in non-responder MDD patients.

The majority of studies that have examined depression have been prospective studies. Our report describes the first naturalistic study examining BDNF levels in remission and non-responder groups. To better understand the role of BDNF in MDD, we compared the changes in plasma BDNF levels in remission and non-responder groups of patients with depressive syndrome.

## Methods

### Depression Assessment

The Montgomery-Åsberg Depression Rating Scale (MADRS) [13] is increasingly employed in clinical research since results from earlier studies had suggested that the scale could be superior to the traditional HAMD17 with respect to sensitivity to change [13], [14], [15] and other psychometric properties [16]. The severity of depression was assessed using the MADRS every two weeks by independent experienced raters. The raters were objective and were not concerned with treatment outcome. We used a MADRS score ≤18 points to separate symptoms more than moderate from symptoms mild depression [17]. We used MADRS scores to identify patients with depressive syndrome (defined as MADRS of at least 18 points), and to differentiate between non-responders (defined as those showing a <50% reduction in MADRS score from the depressive symptom stage) and patients in remission (defined as those with a MADRS score ≤8 after treatment [17]). The period from the depressive syndrome stage to the response stage was 7.2±8.6 weeks, and the period from the depressive syndrome stage to the remission stage was 12.3±12.6 weeks. To investigate differences between the remission and the non-responder groups, we examined different time periods during the course of treatment. The treatment period selected for the non-responder group was closely matched with that of the remission group and was determined to be an 8-week period. Furthermore, the period-matched depressive symptom-remission time frame was determined to be 12 weeks in the non-responder group.

**Figure 1:**
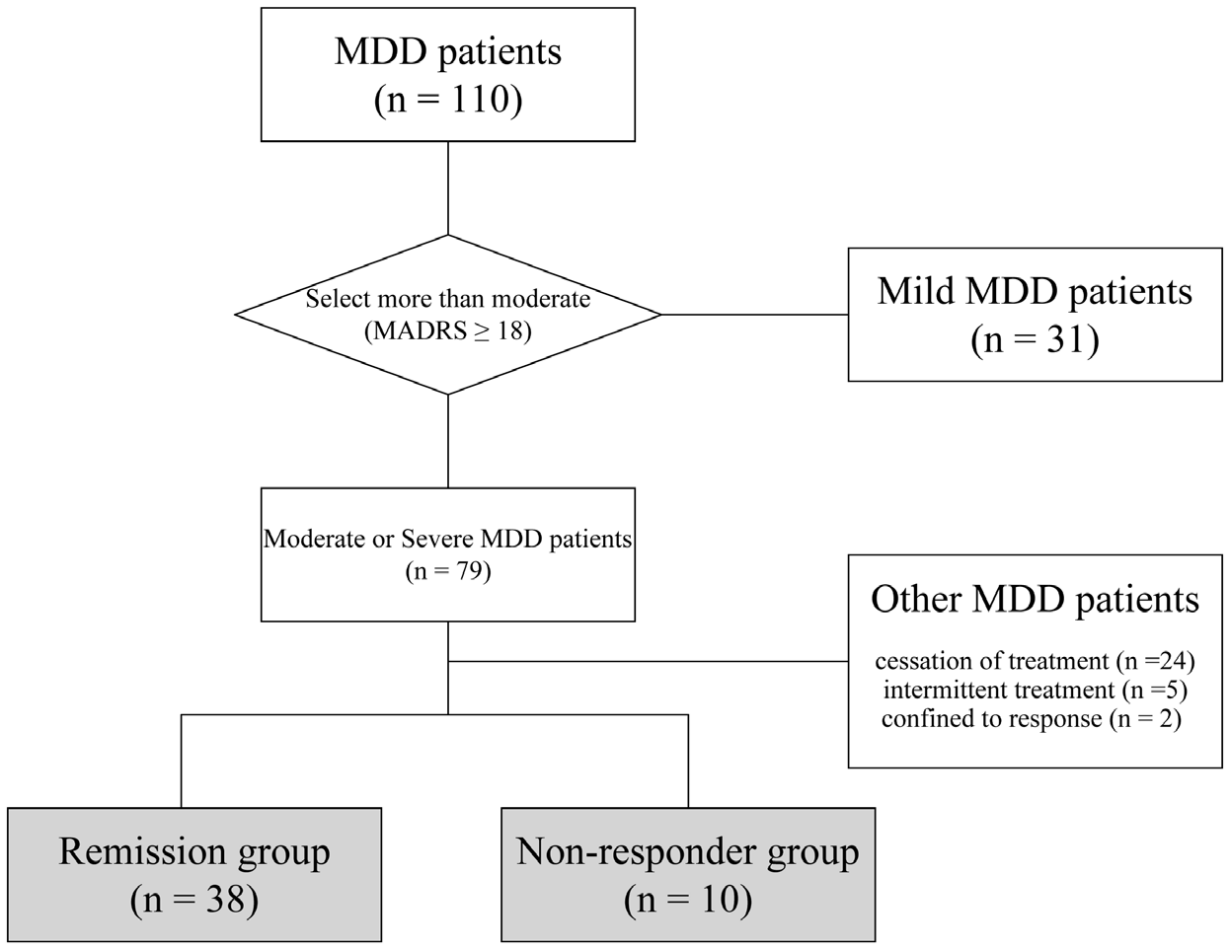
Diagram showing the selection of subjects during the study.

### Subjects

A detailed flow chart of patient selection and grouping during the study is shown in Fig. 1. The subjects were recruited from a total of 110 MDD patients admitted to the in- and out-patient clinics of Sato Hospital, Koutokukai, between June 2006 and March 2009. All patients were suffering from a current major depressive episode–single episode [DSM-IV-TR codes: 296.2] or recurrent episodes [DSM-IV-TR codes: 296.3]–diagnosed according to the Diagnostic and Statistical Manual for Mental Disorders, fourth edition, text version (DSM-IV-TR, American Psychiatric Association, 2000). Subjects with any other diagnosed mental or severe physical illness were excluded from the study. After the procedures were fully explained, all participants gave informed consent. Written informed consent was obtained from all patients participating in the study and the study protocols were approved by the Ethics Committee of Sato Hospital, Koutokukai and the Ethics Committee of Graduate School of Pharmaceutical Sciences, Tohoku University, and was standard procedure in clinical trials involving vulnerable participants in the Japan. This study was performed according to the ethical standards of the Declaration of Helsinki. Also, written informed consent was obtained from the participants, and their parents (guardians) if the participants were children. All participants who declined to participate or otherwise did not participate were eligible for treatment and were not disadvantaged in any other way by not participating in the study.

As a criterion for inclusion of patients in this study, we chose a MADRS score ≥18 points (symptoms more than moderate). Thus, from a total of 110 patients, 79 were selected for inclusion in the study. We then categorized these patients into two groups: a remission group (patients experiencing an improvement of symptoms; MADRS scores ≤8 after treatment) and a non-responder group (patients refractory to treatment, showing a <50% reduction in MADRS score). The final analysis included 48 patients, with 38 subjects in the remission group (19 men and 19 women; average age 44.3±18.6 years, range 17–87 years), and 10 subjects in the non-responder group (three men and seven women; average age 50.4±15.2 years, range 18–71 years). Subjects were divided into two groups based on either sex or median age (43.9 years) to investigate whether these factors affected plasma BDNF. Thirty-one patients were excluded because they either ceased treatment within three months (n = 24), received intermittent treatment (n = 5), or showed response with incomplete remission (n = 2).

None of the subjects was taking hormone therapies (including oral contraceptives). Most patients (47/48 patients) had been prescribed antidepressants by psychiatrists. Psychiatrists treated patients with homogeneous psychotherapy. The following antidepressant drugs were administered to the remission group in the depressive syndrome stage: amitriptyline (50–150 mg/day; n = 2), clomipramine (30–150 mg/day; n = 4), fluvoxamine (25–150 mg/day; n = 11), imipramine (75 mg/day; n = 1), maprotiline (75 mg/day; n = 1), milnacipran (50–200 mg/day; n = 8), paroxetine (10 mg/day; n = 3), sertraline (25–100 mg/day; n = 8), sulpiride (150–300 mg/day; n = 6) and trazodone (25–100 mg/day; n = 4). The non-responder group received the following antidepressant drugs in the depressive syndrome stage: amoxapine (125 mg/day; n = 1), aripiprazole (3 mg/day; n = 1), fluvoxamine (125 mg/day; n = 1), maprotiline (25 mg/day; n = 2), milnacipran (150 mg/day; n = 1), paroxetine (10–40 mg/day; n = 5), sertraline (25–100 mg/day; n = 2), sulpiride (300 mg/day; n = 1) and trazodone (50 mg/day; n = 1). The following antidepressant drugs were administered to the remission group in the response stage: amitriptyline (50–150 mg/day; n = 3), aripiprazole (3 mg/day; n = 1), clomipramine (50–75 mg/day; n = 5), fluvoxamine (50–150 mg/day; n = 9), imipramine (150 mg/day; n = 1), maprotiline (50–75 mg/day; n = 3), milnacipran (50–150 mg/day; n = 8), paroxetine (10–30 mg/day; n = 3), sertraline (25–100 mg/day; n = 9), sulpiride (50–300 mg/day; n = 7) and trazodone (25–100 mg/day; n = 3). The non-responder group received the following antidepressant drugs at the 8-week period: amitriptyline (25 mg/day; n = 1), amoxapine (175 mg/day; n = 1), aripiprazole (18 mg/day; n = 1), clomipramine (105 mg/day; n = 1), hange-kobuku-to (a Chinese herbal medicine; 7.5 g/day; n = 1), imipramine (30 mg/day; n = 1), maprotiline (75 mg/day; n = 1), milnacipran (50–60 mg/day; n = 3), paroxetine (20–40 mg/day; n = 4), sertraline (25 mg/day; n = 1), sulpiride (300 mg/day; n = 1) and trazodone (50 mg/day; n = 1). The following antidepressant drugs were administered to the remission group in the remission stage: amitriptyline (50–150 mg/day; n = 3), aripiprazole (6 mg/day; n = 1), clomipramine (50–100 mg/day; n = 7), fluvoxamine (50–150 mg/day; n = 7), imipramine (250 mg/day; n = 1), maprotiline (75–100 mg/day; n = 2), milnacipran (50–200 mg/day; n = 8), paroxetine (10–30 mg/day; n = 4), sertraline (25–100 mg/day; n = 9), sulpiride (150–300 mg/day; n = 5) and trazodone (25–100 mg/day; n = 3). Meanwhile, the non-responder group received the following antidepressant drugs during the 12-week period: amitriptyline (25 mg/day; n = 1), amoxapine (100–175 mg/day; n = 3), aripiprazole (15 mg/day; n = 1), clomipramine (25–225 mg/day; n = 2), hange-kobuku-to (7.5 g/day; n = 1), maprotiline (75 mg/day; n = 1), milnacipran (50–180 mg/day; n = 3), paroxetine (20–40 mg/day; n = 2), sertraline (25 mg/day; n = 1) and trazodone (50 mg/day; n = 1). Although one subject underwent modified electroconvulsive therapy (weeks 36–41), the patient did not achieve a response. In one patient, the side effects of the antidepressants were considered too adverse; therefore, the patient asked to receive Chinese medicine rather than antidepressants. There was no bias in treatments with SSRIs, SNRIs, TCA and tetracyclic antidepressants between the remission and non-responder groups within the depressive syndrome stage and at the end point (Chi-square test, p>0.05).

### Sample Collection

Blood was withdrawn from each subject by venipuncture into a blood collection tube containing EDTA as an anticoagulant between 10∶00 and 17∶00. The tubes were immediately cooled to 4°C and then centrifuged at 2000×*g* for 20 min. Plasma was kept frozen at –80°C until assayed.

### BDNF Assay

Plasma BDNF levels were measured using an ELISA kit (BDNF Emax Immunoassay System, Promega, Madison, WI, USA) after appropriate dilution of samples (1∶10 to 1∶50) in blocking and sample buffer according to the manufacturer’s instructions. The BDNF standard provided with this system was used to generate a standard curve that was linear between 3.9 and 500 pg/ml. Beyond these limits, BDNF concentrations could not be accurately extrapolated from the standard curve. Therefore, to determine BDNF concentrations in the diluted samples, we used only values that were within the linear range of this standard curve. Briefly, 96-well flat-bottom immunoplates were coated with anti-BDNF monoclonal antibody (mAb) and incubated at 4°C for 18 h. Plates were washed with Tris-buffered saline containing 0.1% Tween 20, pH 7.6 (TBS-T). After blocking non-specific binding with blocking and sample buffer for 1 h at room temperature (RT), standards and samples were added to the plates, incubated on a shaker for 2 h at RT, and then washed with TBS-T. The plates were subsequently incubated with anti-human BDNF polyclonal antibody at RT for 2 hours, washed, and incubated with anti-IgY antibody conjugated to horseradish peroxidase for 1 hour at RT, followed by washing with TBS-T. Tetramethyl-benzidine was then added to produce the color reaction. After stopping the reaction with 1 N HCl, the absorbance was read at 450 nm on a Sunrise Classic microplate reader (Tecan, Mannedolf, Switzerland) and BDNF concentrations were determined automatically according to the BDNF standard curve (ranging from 7.8 to 500 pg/ml of BDNF). Measurements were performed in duplicate.

### Data Analysis

Statistical analyses of MADRS scores and plasma BDNF levels were performed using one-way repeated measures analysis of variance (rep-ANOVA) with three levels of symptoms or periods. Post-hoc tests were performed on ANOVA results using the Bonferroni correction for multiple comparisons. Between-group comparisons were performed using unpaired, two-tailed Student’s t-tests. Data are shown as the means ± standard deviation (mean ± S.D.). Correlation analysis was performed using Spearman’s correlation. Statistical significance was defined as *p*<0.05. Normality testing was performed using the Shapiro-Wilk test. Possible violations of the sphericity assumption were assessed by Mauchly’s test. Analyses were performed using SPSS software version 16.0.

## Results

### Characteristics of MDD Patients

The subjects’ characteristics are summarized in Table 1. No significant differences were found between the remission and non-responder groups in terms of gender, age or MADRS score in the depressive syndrome stage.

**Table 1:** Initial Characteristics of Remission and Non-responder groups among MDD patients.

		Remission group	Non-responder group	p-value
		(n = 38)	(n = 10)	
Gender (M/F)		19/19	3/7	0.259^a^
Mean age (S.D.)		44.3 (18.6)	50.4 (15.2)	0.178^b^
Mean MADRS score (S.D.)		33.7 (8.9)	35.1 (6.5)	0.454^b^
Mean plasma BDNF (S.D.) (pg/mL)	1827 (1340)		2932 (2373)	0.002^b^

MADRS, Montgomery-Åsberg Depression Rating Scale; BDNF, brain-derived neurotrophic factor.

a Chi square test.

b Student’s t-test.

### Remission Group

Remission group patients were defined as those with a MADRS score ≤8, reducing from a score of at least 18 points, after treatment. The MADRS scores before treatment and at the time of response and remission after treatment were 33.7±8.9, 10.9±5.9, and 5.0±2.4, respectively. Patients in the remission group had significantly reduced MADRS scores during the treatment (repeated-measures ANOVA; F_1, 37_ = 344.017, *p*<0.001). There were significant differences in MADRS scores among stages within the remission group (depressive symptoms vs response (*p*<0.001), depressive symptoms vs remission (*p*<0.001), and response vs remission (p<0.001); Bonferroni’s multiple comparison).

**Figure 2:**
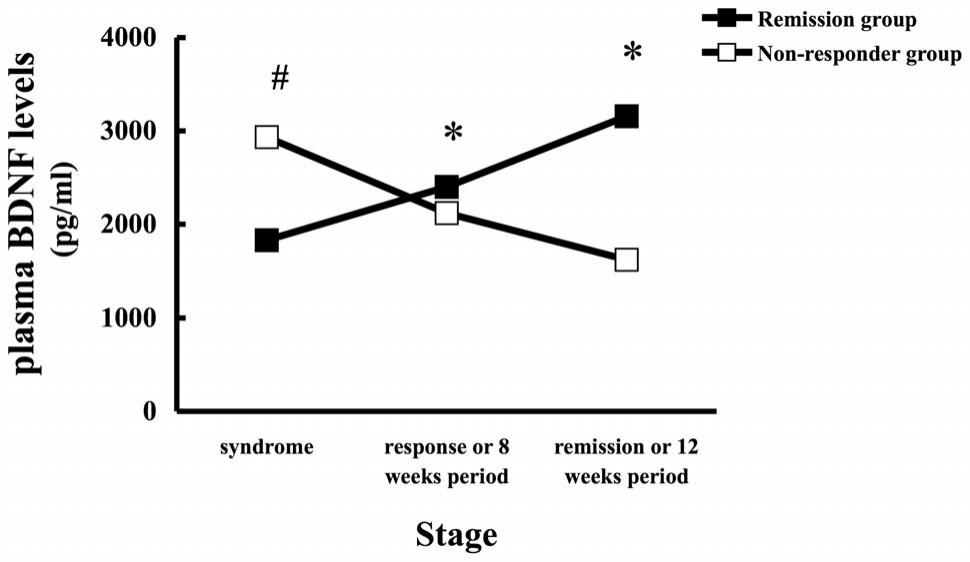
Changes in plasma BDNF levels in MDD patients (remission [▪, n = 38] and non-responder [□, n = 10] groups). The period-matched symptom-response and symptom-remission outcomes were examined at 8 and 12 weeks, respectively, in the non-responder group. Plasma BDNF levels were measured by immunoassay. Each point represents the mean. The statistical significance of differences was calculated using repeated-measures ANOVA with *post-hoc* Bonferroni testing (*p<0.05). The statistical significance of differences in plasma BDNF levels between remission (▪) and non-responder (□) groups at the depressive syndrome stage were calculated using the Students’ t-test (^#^ *p*<0.05).

The period from the depressive syndrome stage to the response stage was 7.2±8.6 weeks, and the period from the depressive syndrome stage to the remission stage was 12.3±12.6 weeks. Plasma BDNF levels in the depressive syndrome, response and remission stages in the remission group were 1 827±1 340, 2 402±1 610, and 3 158±2 033 pg/ml, respectively. Patients in the remission group had significantly higher plasma BDNF levels at remission than in the depressive syndrome and response stages (repeated-measures ANOVA; F_1, 37_ = 25.083, *p*<0.001) (Fig.2). Plasma BDNF levels differed significantly among stages within the remission group (depressive symptoms vs response (*p* = 0.004); depressive symptoms vs remission (*p*<0.001); and response vs remission (*p* = 0.003); post hoc ANOVA Bonferroni’s Multiple Comparison).

**Figure 3:**
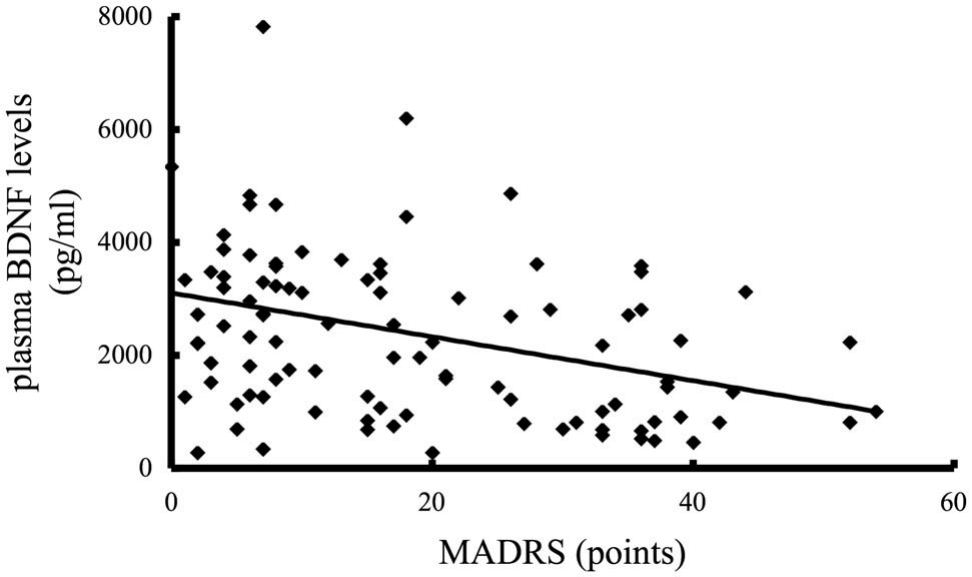
Correlation of plasma BDNF levels and MADRS scores in the remission group (n = 38*, 3 states). There was a significant negative correlation between plasma BDNF levels and MADRS scores (ρ = –0.287, *p* = 0.003). Statistical analysis was performed using Spearman’s correlation test.

Treatment of the remission group led to an expected decrease in MADRS scores, and this was accompanied by a significant increase in plasma BDNF levels. Correspondingly, we found a significant negative correlation between MADRS scores and plasma BDNF levels within the remission group (ρ = –0.287, *p* = 0.003, n = 114) (Fig.3).

### Non-responder Group

To investigate differences between the remission and the non-responder groups, we examined different time periods during the course of treatment.

The MADRS scores in the non-responder group at the depressive syndrome stage, and at 8 and 12 weeks after the commencement of treatment were 35.1±6.5, 25.8±7.7, and 35.2±11.4, respectively. Patients in the non-responder group exhibited no significant change in MADRS score between 8 and 12 weeks (repeated-measures ANOVA; F_1, 9_ = 0.001, *p* = 0.982).

Plasma BDNF levels in the non-responder group at the depressive syndrome stage, and at 8 and 12 weeks after the commencement of treatment were 2 932±2 373, 2 117±2 042, and 1 619±1 698 pg/ml, respectively. Interestingly, patients in the non-responder group showed significantly reduced plasma BDNF levels between weeks 8 and 12 (repeated-measures ANOVA; F_1, 9_ = 6.743, *p* = 0.029) (Fig.2). Surprisingly, treatment of the non-responder group produced no change in MADRS score by 12 weeks, although these patients did show a significant decrease in plasma BDNF levels. We found no significant correlation between MADRS scores and plasma BDNF levels (ρ = –0.112, p = 0.554, n = 30) in the non-responder group.

### Comparison of the Remission and Non-responder Groups at the Depressive Syndrome Stage

To determine whether plasma BDNF levels could predict treatment outcome, we examined plasma BDNF levels in the remission and non-responder groups at the initial depressive syndrome stage. Surprisingly, the non-responder group had higher plasma BDNF levels compared with the remission group (*p* = 0.002) (Fig.2).

### No Effect of Sex or Age on Plasma BDNF

We next investigated whether sex and/or age affected plasma BDNF. We divided the subjects into two groups based on either sex or median age (43.9 years). The results for the remission group are summarized in Table 2. Plasma BDNF was not affected by sex or age.

**Table 2:** MDD Remission group characteristics by sex and age, and Non-responder group characteristics.

				Syndrome			Response			Remission		F	p-value^a^
Total Remission													
(n = 38)	MADRS (S.D.)		33.7		(8.9)	10.9		(5.9)	5		(2.4)	334	<0.001
	BDNF (S.D.)		1827		(1340)	2402		(1610)	3158		(2033)	25.1	<0.001
Male Remission		(mean age, 36.9 (14.0)^b^)											
(n = 19)	MADRS (S.D.)		33.1		(8.9)	11.2		(6.2)	5		(2.3)	155.3	<0.001
	BDNF (S.D.)		1811		(1309)	2239		(1173)	2994		(2042)	8.85	0.008
Female Remission		(mean age, 51.7 (19.9)^b^)											
(n = 19)	MADRS (S.D.)		34.3		(9.1)	10.7		(5.9)	5.1		(2.6)	181.9	<0.001
	BDNF (S.D.)		1843		(1405)	2565		(1973)	3321		(2067)	16.87	0.001
Young Remission		(median age <43.9; mean age, 28.7 (6.9))											
(n = 19)	MADRS (S.D.)		34.6		(8.7)	12.6		(6.6)	5.5		(2.1)	180.5	<0.001
	BDNF (S.D.)		1428		(1163)	1995		(1210)	2582		(1419)	10.89	0.004
Old Remission		(median age ≥43.9; mean age, 60.0 (12.0))											
(n = 19)	MADRS (S.D.)		32.7		(9.2)	9.3		(4.8)	4.6		(2.7)	156.2	<0.001
	BDNF (S.D.)		225.8		(1414)	2810		(1873)	3733		(2405)	13.82	0.002
				**Syndrome**			**8 weeks**			**12 weeks**		**F**	**p-value** a
Non-responder													
(n = 10)	MADRS (S.D.)		35.1		(6.5)	25.8		(7.7)	35.2		(11.4)	0.001	0.982
	BDNF (S.D.)		2932		(2373)	2117		(2042)	1619		(1698)	6.743	0.029

MADRS, Montgomery-Åsberg Depression Rating Scale; BDNF, brain-derived neurotrophic factor.

a one-way repeated measures analysis of variance.

b significant difference between male and female ages by Student’s t-test.

## Discussion

To gain insight into the different outcomes during the course of treatment for depression, we examined whether plasma BDNF levels underwent a change at different stages (syndrome, response and remission) and the equivalent time points in non-responders, focusing on differences between remission and non-responder groups. In the remission group, plasma BDNF levels increased significantly with clinical improvement, independent of sex and age.

Although options for pharmacologic treatment have expanded significantly in the past 20 years, between one- and two-thirds of patients do not respond to the first antidepressant prescribed, and 15–33% do not respond to multiple interventions [18]. In this study, the non-responder group comprised 21% (10/48) of the study population, consistent with the above report. However, the resultant difference in the numbers of patients in the remission (79% (38/48)) and non-responder groups (21% (10/48)) is a limitation of a naturalistic study such as ours.

Of note, we show for the first time that patients in the non-responder group have significantly decreased plasma BDNF levels during the syndrome’s 8–12 week period. Thus, plasma BDNF may serve as an important biomarker for the prognosis of MDD.

In this study, we focused on remission. An advantage of a naturalistic study such as ours is that we could compare plasma BDNF as a biomarker between patients achieving remission and non-responders. For the purposes of comparison, we observed scores in non-responders at the corresponding time points to those at which patients in the remission achieved a response and remission. However, the disagreement of the period of remission and the period examined in non-responders (despite each stage being matched) is a limitation of naturalistic studies. Another limitation is the potential variation in drug treatment between the two groups of depressed patients. The effects of antidepressants on peripheral BDNF levels are not uniform [19], [20]. Thus, although most studies to date have shown that serum BDNF levels increase with antidepressant treatment, different classes of antidepressants-induced changes in BDNF in the peripheral blood are not always uniform. However, in the present naturalistic study, there was no major bias in drug treatment between the remission and non-responder groups. There was no bias in treatments with SSRIs, SNRIs, TCA and tetracyclic antidepressants between the remission and non-responder groups within the depressive syndrome stage and at the end point (Chi-square test, p>0.05). Also, some patients were treated with multiple antidepressants.

Individual differences in plasma BDNF levels were large, and consequently, plasma BDNF levels may have differed between groups. Therefore, although plasma BDNF may be a valuable biomarker for the treatment of depression, it may not be appropriate or feasible to establish a normal range (as is done for numerous other serum components to establish a baseline for assessing physiological status). Thus, it is necessary to measure plasma BDNF regularly in each MDD patient, and a careful examination of the BDNF profile, to examine trends or shifts, is necessary for the clinician to select an appropriate treatment.

Plasma BDNF levels decreased during the course of treatment in the non-responder group. BDNF levels are increased by not only antidepressants, but also by environmental enrichment [21] and modest exercise [22]. In contrast, BDNF levels are decreased by stressful events. Plasma BDNF levels were decreased in non-responders treated with antidepressants suggesting that the ability of stress to decrease BDNF levels may be greater than the ability of antidepressants to increase BDNF levels.

Notably, we found that plasma BDNF concentrations were significantly different between the remission and non-responder groups at the depressive syndrome stage. This important observation suggests that the biological backgrounds of patients with treatment-responsive MDD and patients with treatment-resistant MDD might differ, and that high plasma BDNF levels during the depressive syndrome stage may be indicative of treatment-resistant MDD patients. Thus, plasma BDNF levels may help the clinician to predict clinical outcome. In particular, if plasma BDNF levels decrease or are unchanged in an individual with regularly measured plasma BDNF, the clinician may need to reevaluate treatment strategy.

In 2002, the involvement of serum BDNF in stress and major depression was reported for the first time [5]. Over the last 14 years, most studies have examined serum BDNF rather than plasma BDNF [23]. However, Piccinni et al suggested that plasma BDNF may be more appropriate as a biomarker of physiological status, while serum BDNF is more likely to represent a trait marker [7]. Very recently, plasma BDNF was associated with response in the early course of treatment for depression [24].

In our study, plasma BDNF levels in patients in the remission group significantly increased during the transition from syndrome to response to remission stages, suggesting that plasma BDNF may be a useful marker of physiological status and that it should be examined in patients on a regular basis.

A correlation between cortical BDNF and serum BDNF in young rats was first shown by Karege et al. [5]. In contrast, Elfving et al. found a negative correlation between hippocampal and serum BDNF levels [25]. In blood, BDNF is mainly stored in thrombocytes, with only a minor free fraction present in plasma [26]. Recently, plasma BDNF levels were shown to be positively correlated with hippocampal BDNF levels [27]. The origin of plasma BDNF is not entirely clear, although it appears that the hippocampus is the main source.

Brain imaging studies have documented a reduction in hippocampal volume in depressed subjects [28], which can be attenuated [29], or even improve [30] with antidepressant treatment. These observations suggest that plasma BDNF may be involved in the pathophysiology of MDD. Thus, we posit that increased plasma BDNF may have a therapeutic effect on the hippocampus.

Six of the 38 patients (16%) in the remission group exhibited a reduction in plasma BDNF levels. One possible explanation for this is that, although depression was diagnosed according to DSM-IV-TR, differences in biological backgrounds may generate different subgroups within the remission group. Another possibility is that an improvement in depressive symptoms may be directly due to an effect of increased monoamine levels produced by the antidepressant, although this hypothesis needs to be examined. These findings suggest that plasma BDNF levels are likely to be a biomarker for MDD, and that the onset and improvement of the disease might be associated with changes in plasma BDNF levels elicited by antidepressant treatment.

In summary, the present study shows that plasma BDNF levels are positively correlated with clinical improvement in patients who undergo remission, and that patients who are refractory to treatment have higher plasma BDNF levels than patients who achieve remission at the initial depressive syndrome stage. Therefore, it is very likely that plasma BDNF levels play an important role in MDD. Our naturalistic preliminary study reveals that plasma BDNF could represent a useful biomarker for predicting clinical outcome during the course of treatment for MDD.
